# Green Biosynthesis of Silver Nanoparticles from *Moringa oleifera* Leaves and Its Antimicrobial and Cytotoxicity Activities

**DOI:** 10.1155/2022/4136641

**Published:** 2022-09-19

**Authors:** Gufran Mahmood Mohammed, Sumaiya Naeema Hawar

**Affiliations:** Biology Department, College of Education for Pure Sciences/Ibn Al-Haitham, University of Baghdad, Iraq

## Abstract

The plant occupied the largest area in the biosynthesis of silver nanoparticles, especially the medicinal plants, and it has shown great potential in biotechnology applications. In this study, green synthesis of silver nanoparticles from *Moringa oleifera* leaves extract and its antifungal and antitumor activities were investigated. The formation of silver nanoparticles was observed after 1 hour of preparation color changing. The ultraviolet and visible spectrum, Fourier transform infrared spectroscopy, X-ray diffraction, scanning electron microscopy, and transmission electron microscopy techniques were used to characterize synthesis particles. Ultraviolet and visible spectroscopy showed a silver surface plasmon resonance band at 434 nm. Fourier transform infrared analysis shows the possible interactions between silver and bioactive molecules in *Moringa oleifera* leaves extracts, which may be responsible for the synthesis and stabilization of silver nanoparticles. X-ray diffraction showed that the particles were a semicubic crystal structure and with a size of 38.495 nm. Scanning electron microscopy imaging shows that the atoms are spherical in shape and the average size is 17 nm. The transmission electron microscopy image demonstrated that AgNPs were spherical and semispherical particles with an average of (50–60) nm. The nanoparticles also showed potent antimicrobial activity against pathogenic bacteria and fungi using the well diffusion method. *Candida glabrata* found that the concentration of 1000 *μ*g/mL exhibited the highest inhibition. As for bacteria, the concentration of 1000 *μ*g/mL appeared to be the inhibition against *Staphylococcus aureus*. *Moringa oleifera* AgNPs inhibited human melanoma cells A375 line significant concentration-dependent cytotoxic effects. The powerful bioactivity of the green synthesized silver nanoparticles from medical plants recommends their biomedical use as antimicrobial as well as cytotoxic agents.

## 1. Introduction

Nanotechnology is a well-known field of research since the last century. The prefix (nano) refers to the Greek word which means dwarf or small thing. Also, nanoscience is the study of structures and molecules on the nanometer scale. While nanotechnology means the equipment used in practical applications [1]. The origin of nano development can be traced back to the 15th century BC when scientists investigated the question of whether the matter could be down into smaller parts and whether the formation of microparticles was indivisible [2]. Nanometallices entered various fields such as environment, medicine, and engineering, and it is divided according to the method of its synthesis, which may be physical, chemical, or biological. The first two methods may have unintended effects such as environmental pollution and high energy consumption [3]. The biological synthesis method is safer, more useful, and less expensive. Also, there are many metals used such as gold, silver, lead, zinc oxide, copper, and others [4], and silver nanoparticles are the most promising. It has been observed that silver nanoparticles do not affect living cells, so they cannot provoke microbial resistance [5], and silver nanoparticles have strong antimicrobial activity [6]. Studies mentioned green biosynthesis from bacteria [7], fungi [8], and plants [9]. The method of biosynthesis from plants is more efficient and requires little cost and maintenance, while using other organisms need a specific medium and a certain period [10]. Medical plants have occupied an essential role in the lives of people all over the world, starting with ancient Indian Ayurvedic and traditional medicine. Several medicinal plants have been used in the green biosynthesis of silver nanoparticles such as *Thymus vulgaris*, *Metha piperita*, *Zingber officinale* [11], *Alpinia officinarum* [12], *Teucrium polium* [13], and *Prangos ferulacea* [14]. The plant phytochemicals or secondary metabolites such as proteins, polyphenols, phenolic acids, ketones, terpenoids, and amides constantly play a significant role in the biosynthesis of nanoparticles [15]. *Moringa* belongs to the Family Moringaceae and the order Capparales [16]. *Moringa* contains 13 species and is called by many common names according to the growing regions. *Moringa* has been used in the biosynthesis of nanoparticles by a number of researchers [17]. Investigating the efficiency against *Candida albicans*, the study compared the effects of aqueous leaf extract with an ointment containing silver nanoparticles, synthesized and used as an antimicrobial agent [18]. Also, [19] mentioned the ecologically friendly manufacture of silver nanoparticles from fresh leaves of the *Moringa oleifera* plant and evaluated against *Escherichia coli*, *Klebsiella pneumonia*, *Staphylococcus aureus,* and *Bacillus subtilis*. *Moringa* contains a long list of medical use including antibacterial [20], antifungal [21], antiparasitic [22], antiviral [23], wound healing, antimicrobial, antioxidant, and proliferative properties [24]. In the present study, we evaluated the effectiveness of the synthesis of silver nanoparticles from aqueous extract of *Moringa oleifera* leaves extract against the clinical strain of bacteria (positive and negative Gram) and human pathogenic fungi (*Candida* species)and evaluated the cytotoxicity effect of silver nanoparticles on human skin cancer.

## 2. Materials and Methods

### 2.1. Materials

The chemicals silver nitrate, Sabouraud dextrose agar, and Muller Hinton agar were purchased from BDH, England. Tissue culture plastic wares were obtained from BD Bioscience, USA. 2,2-Diphenyl-1-picrylhydrazyl (DPPH) and 3-(4,5-dimethylthiazal-z-yl)-2,5-diphenylterazolium (MTT) were purchased from Sigma Aldrich, UK. A375 and WRL68 cell lines were purchased from the Department of Pharmacy/College of Medicine, University of Malaya.

### 2.2. Organisms

Clinical strains like *Candida albicans*, *Candida tropicalis*, *Candida glabrata*, *Staphylococcus aureus* (Gram-positive), *Escherichia coli*, (Gram-negative), and *Klebsiella pneumonia* (Gram-negative) were obtained from a hospital in Baghdad Governorate for patients and diagnosed in the hospital laboratories. Then, it was preserved in the PDA in a refrigerator at 4°C in the Advanced Fungi Laboratory, Department of Biology/College of Education for Pure Sciences (Ibn Al-Haitham)/University of Baghdad.

### 2.3. Preparation of Leaves Aqueous Extract

*M. oelifera* healthy and green leaves were collected from the green belt surrounding Hilla City, in the south of Baghdad. The leaves were washed with tap water three times and dried using sterile filter paper and the unimportant parts were removed. After drying, it is ground using an electric grinder (WAHL, USA) and then stored in sealed and labeled bags until used. [25]. The method was followed with some modifications, where 100 g of plant leaves powder was taken and mixed with 1000 mL of distilled water, heated for two hours at a temperature of 50°C, and placed in a centrifuge (Hettich, Germany). The supernatant was taken and placed in a rotary evaporator (IKA, England) to collect the dry extract to prepare the nanosolution.

### 2.4. Biosynthesis AgNPs Using *M. oleifera* Leaves Extract

A modified method of [26] was followed, where 1 mM of silver nitrate was prepared at 80°C, and the plant extract was distilled (7 g of the dried plant extract prepared in the previous paragraph into 100 distilled nonionic water) on top it for an hour and in a ratio of silver nitrate solution 80: plant extract 20 (V/V) and pH = 7. Then, it was observed that the color change started and was kept in the dark for five days until the color stabilized; after that, it was purified and dried according to [27]. The concentration of silver (Ag) in the nanosolution was measured by atomic absorption spectroscopy (SHIMADZU, Japan) at the Ministry of Science and Technology.

### 2.5. Characterization of Silver Nanoparticles

Ultraviolet and visible spectroscopy analyses were performed to determine *λ*max by using UV-Vis spectroscopy (UV-1650 PC meter, Japan) from 250 to 1100 nm at a resolution of 1 nm [28]. Fourier transform infrared spectroscopy (FTIR) analysis was carried out using an FTIR spectrometer (Spectrum TWO N, PerkinElmer's, USA) in attenuated total reflection mode and spectral range 500–4000 cm^−1^ with a resolution of 4 cm^−1^ [29]. Various Bragg's reflections were observed in the XRD pattern in the wide angle ranging from 20 to 80. Nanoparticle size is calculated by Scherrer's equation: D = K *λ*/ß cos *θ*, where D is the average crystal size, *K* = Scherrer's constant 0.89, *ß* = full-width half maximum (FWHM) 0.52000, and *θ* = diffraction angle (Bragg angle) 38.164/2 [30]. Scanning electron microscopy (SEM) analysis was conducted using the TESCAN MIRA3 device (FRENCH) instrument to determine the morphology (size and shape) of synthesized nanoparticles [31]. Transmission electron microscopy (TEM) analysis was used to study the shape of the nanoparticles conducted using a TEM (EM10C, Germany) device at a voltage of 100 KV [32].

### 2.6. Evaluation of Antimicrobial Activity

The silver nanoparticles synthesized using *M. oleifera* leaves extract were tested for their antipathogenic activity against pathogenic fungi (yeast) and bacteria by the agar well diffusion method. The pure cultures of fungi were subcultured on SDA. Each strain was swabbed uniformly onto the individual plates, using sterile cotton swabs. For fungi, wells of 5 mm diameter containing the prepared nanoparticle solution at concentration of 1000, 800, and 600 *μ*g/mL were put on SDA and incubated at 37°C for 48 h. For bacteria, wells with a diameter of 5 mm were made on Muller Hinton agar plates and incubated at 37°C for 24 h. Silver nitrate with 1 mM was used as the positive control and the aqueous extract of *M. oleifera* leaves as a negative control. For each experiment, the diameter of zone inhibition was measured in millimeters and was recorded as the mean ± SE of the triplicate experiment [33].

### 2.7. Evaluation of Cytotoxic Activity

Human melanoma cells A375 (isolated from a 54-year-old female tumor) and normal WRL 68 cells (derived from cervical tissue cells) were used to assess the cytotoxic effect of synthesized *Moringa oleifera* leaf-silver NPs. Ethical approval was obtained by the University of Baghdad' Ethics Committee, NO. 6255 at 24/10/2021.

#### 2.7.1. MTT Assay

3-(4, 5-Dimethylthiazol-z-yl)-2,5-diphenyltetrazolium (MTT) was used to evaluate the cytotoxicity of the prepared extract and nanoparticle solution. Cells were cultivated in 96-well plates at a density of 1 × 10^−4^ cells/well for 24 hours incubation at CO_2_ 5% and 37°C. Following this, various doses of synthesized nanosolution (12.5, 25, 50, 100, 200, and 400 *μ*l/mL) were added to the culture mix and incubated for another 24 hours; cells cultured with media alone served as the negative control. Twenty microliters of MTT solution (5 mg/mL PBS) were added to each well and incubated for 1 hour. After the medium was removed, 200 *μ*l of DMSO was added to each well, and the plates were incubated at 37°C for 30 minutes. The absorbance was measured at 570 nm, and the percentage of cell growth viability was determined using the following equation.

(1) $$\begin{array}{c} \text{Cell viability}\%=\frac{\text{OD test}}{\text{OD control}}\times 100\text{. } \end{array}$$

### 2.8. Statistical Analysis

The mean ± S.E was calculated for three replicates of the experiment to find out the *p* value using the IBM SSPS program (V.23). One-way ANOVA (LSD) significance is considered at *p* < 0.05.

## 3. Results and Discussion

**Figure 1:**
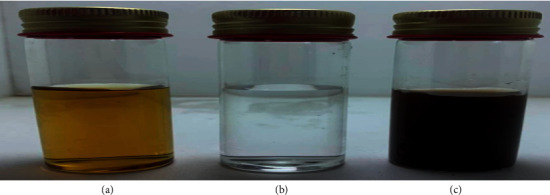
The color changing of *Moringa oleifera* leaves extracts from yellow to dark reddish brown. (a) Leaves extract solution. (b) AgNO_3_ only. (c) Leaves extract after reaction with 1 mM AgNO_3_ solution at 25°C for 5 days.

**Figure 2:**
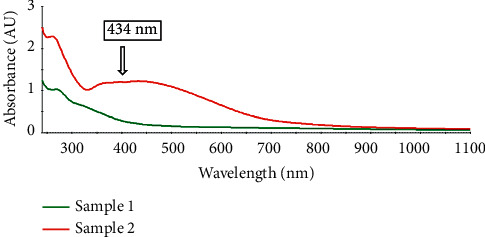
UV-Vis absorption spectrum of *M. oleifera* leaves extract (green color line) and AgNPs (red color line). The absorption spectrum was recorded at room temperature after 5 days of the preparation period.

The first characteristic indicator of silver nanoparticle synthesis is the color change from light yellow to dark reddish-brown (Figure 1). The absorbance of the plant extract and AgNPs nanoparticles solution was measured by UV-Vis spectroscopy; the results found that the maximum absorbance of the *M. oleifera* leaves extract was 268 nm, while the absorbance for the silver nanoparticles solution was 434 nm (Figure 2). The color change indicates the occurrence of surface plasmon resonance in the metal nanoparticles (SPR) [34]; the color change was observed after the leaves extract was incubated with silver nitrate solution and this is evidence of silver ion reduction to silver nanoparticles. Silver nanoparticles synthesized from *Moringa* leaves extract showed SPR peak at 430–440 nm [17, 35], and the results suggested that the extract solution containing reducing compounds plays a significant role in the conversion of silver ions into AgO nanoparticles. Another result of [36] was that synthesis of AgNPs by using *E. hybrida* explains that the color change happened because the leaf extract contains terpenoids and flavonoids that might be the surface-active molecules stabilizing the nanoparticles.

**Figure 3:**
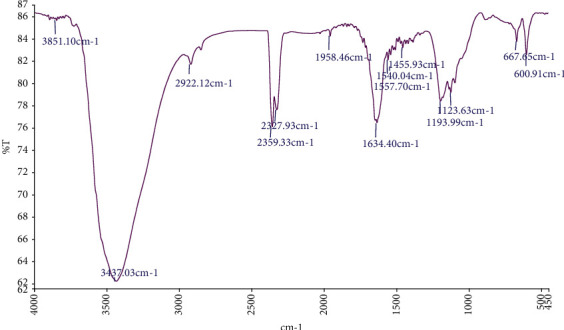
FTIR of the spectrum of green synthesized silver nanoparticles by *M. oleifera* leaves extracts.

An FTIR spectral study was conducted to determine the potential interaction between silver and biologically active molecules, which may be responsible for the synthesis of AgNPs and stability. The results of the FTIR analysis are shown in figure 3, and the FTIR peaks of silver nanoparticles appeared at 1123.63, 1193.99, 1557.70, 1634.40, 2327.93, 2359.33, 2922.12, and 3437.03. The peaks that appear about 600–1400 refer to C–O- carboxyl groups or the C-N group, which is an extension of the amide bonds in proteins [37], and this indicates the high content of proteins in the plant leaf. The peak 1557.70 refers to the nitriles group, which is the organic acid that contains the C≡N-group, which indicates a triple bond between carbon and nitrogen. While the peak at 1634.40 refers to the -C-O and -C≡C- bond, which is primary and secondary fission; whereas, the peaks at 2327.93 and 2359.33 refer to the C=N function. The peak 2922.12 refers to the C-H alkaline group. While, the peak 3437.03 indicates the presence of the O-H bond of phenolic compounds. The presence of phenols and proteins does not only work as a reducing factor but also can act as a stabilizing factor and may prevent clustering by linking to AgNPs through free amino groups or cysteine residues. *Moringa* leaf extract contains phenols, carboxylic acid, proteins, and terpenoids, which are probably responsible for the synthesis and reduction of nanoparticles [38].

**Figure 4:**
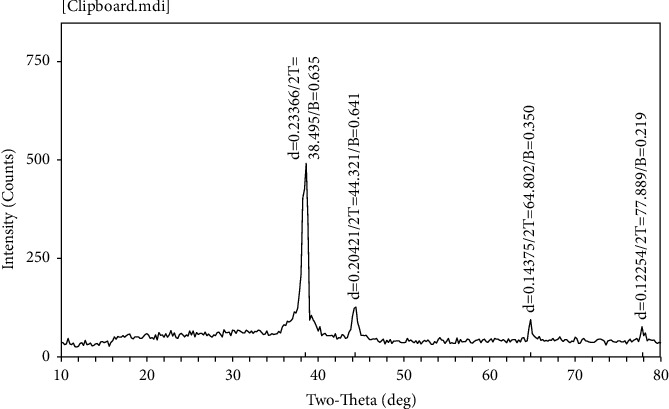
XRD pattern formed after reaction of *M. oleifera* leaves extracts.

The XRD technique was used to determine the crystal structure and particle size of the nanoparticles. The results (Figure 4) showed the formation of a semicubic crystal structure with diffraction angles 38.495, 44.321, 64,802, and 77.889, which correspond to the crystal levels at the Braggs reflection patterns at 2*θ*, and these patterns correspond to (111), (200), (220), and (311). The diffraction peaks were compared with the standard data (JCPDS file No. 04–0783), which showed that the face-centered cubic (FCC) of the silver nanoparticles solution and the crystal size was estimated according to the Debye–Scherrer equation, and this result agrees with the findings of [39] study.

**Figure 5:**
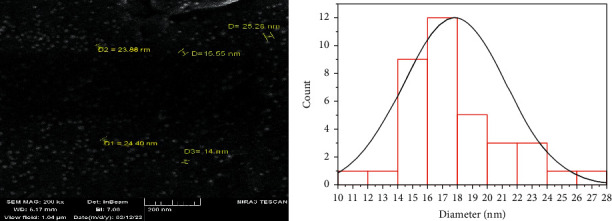
(a) SEM image showing AgNPs at the scale of 200 nm and (b) the size distribution of green synthesized silver nanoparticles.

The SEM analysis was performed to identify the uniformity and surface morphology of AgNPs. The image of the SEM microscope with a size of 200 nm clearly shows that the atoms are spherical in shape and Figure 5(a) shows the size distribution of AgNPs with a diameter in the range of 10–28 nm, so the result showed that the synthesis silver nanoparticles solution was stable and showed no agglomeration of nanoparticles. A previous study by Irfan et al. [40] recorded particles with 50 nm size obtained from the extract of *M. oleifera* gum with silver nitrate, while [41] obtained spherical particles from the extract of *Moringa* leaves with (1 mM) silver nitrate.

**Figure 6:**
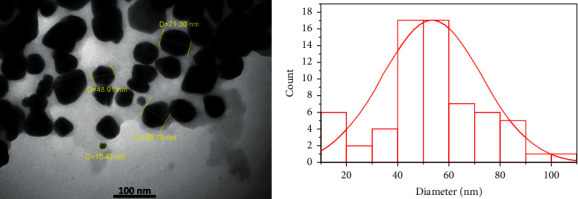
(a) TEM image showing spherical nanoparticles of AgNPs at the scale of 100 nm and (b) the size distribution of green synthesized silver nanoparticles.

The result obtained from the transmission electron microscope (TEM) shows the dispersity, size distribution, and morphology of synthesized AgNPs. Most of the nanoparticles were spherical or near-spherical, and several ones were irregular or polygonal shaped (Figure 6(a)). The size of AgNPs measured by ImageJ was in the range of 10–100 nm, and the average size was about 50–60 nm (Figure 6(b)). This is in agreement with the findings of [35, 42] studies, in which they obtained particles with size of 57 nm from *M. oleifera* leaves extract with silver nitrate. In contrast, [43] observed particles with 45 nm from *Moringa* leaves extract with iron oxide.

**Figure 7:**
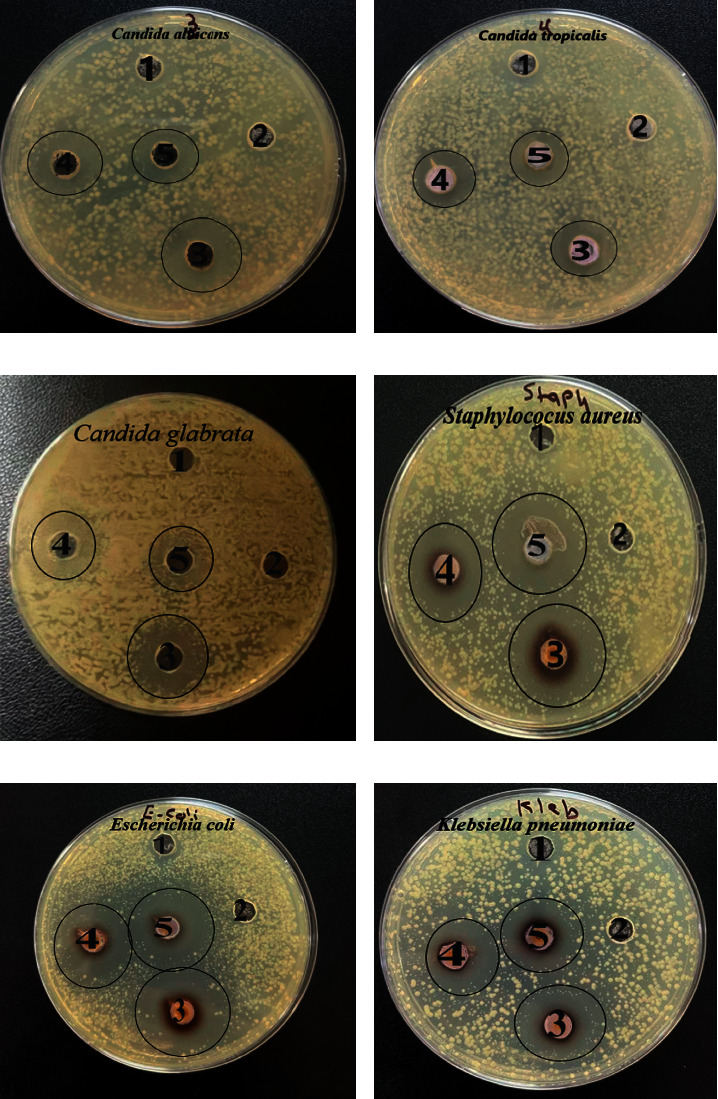
The antimicrobial activity assay against some pathogens. (a)–(c) *Candida* (yeasts). (d)–(f) Different bacteria, using the well diffusion method. (1) AgNO_3_. (2) Plant extract. (3) 1000 *μ*g/mL. (4) 800 *μ*g/mL. (5) 600 *μ*g/mL.

The biological activity results of AgNPs at a concentration of 1000, 800, and 600 *μ*g/mL by using the agar diffusion method on three species of *Candida* (yeasts) and three species of bacteria showed their high activity as shown in Figure 7 and Table 1. The concentration of 10000 *μ*g/mL showed the highest inhibiting effect for *Candida glabrata* with a diameter zone of 18.0 ± 1.0 mm, whereas the lowest inhibition was observed at 800 *μ*g/mL against *C. tropicalis* with a diameter zone of 11.3 ± 0.3 mm. For bacteria, the silver nanoparticles had a greater effect, where the concentration of 1000 *μ*g/mL showed the highest inhibiting effect on *S. aureus* growth in which the diameter of the inhibition zone was 20.0 ± 0.5 mm and against *E. coli* with 19.0 ± 0.5 mm, and for *K. pneumonia* was the lowest effect with 14.6 ± 0.6 mm. In contrast, at the concentration of 600 *μ*g/mL, the highest inhibiting effect was found on *E. coli* growth with a diameter of 21.0 ± 0.5 mm and the lowest effect for *C. glabrata* with an inhibition diameter of 11.6 ± 0.5 mm. No significant inhibition of control has been observed after treatment with 1 mM AgNO_3_ alone.

Silver nanoparticles synthesized from *Moringa* leaf extract exhibit high inhibition on the growth of *S. aureus* and *C. tropicalis*, while the low inhibition rate was against *K. pneumoniae* and *C. krusei* [35]. Furthermore, the observation of [17] study was that the biological activity of silver nanoparticles from *Moringa* leaves extract against *C. albicans* after comparison with the antifungal cream clotrimazole was determined and found that the inhibition is 19–28 mm, while it was 30 mm after using clotrimazole. The antimicrobial activity of silver nanoparticles synthesized by *M. oleifera* seed oil extract has been reported against several pathogenic such as *K. pneumonia, S. aureus, E. coli,* and *C. albicans* [44]. Silver nanoparticles (AgNPs) were synthesized using pu-erh tea leaves extract [45]; this study indicates that AgNPs exhibit strong antimicrobial activity against *Escherichia coli*, *Salmonella typhimurium, Klebsiella pneumoniae*, and *Salmonella enteritidis*. Reference [46] used ribose and sodium dodecyl sulfate (SDS) to green synthesize AgNPs and showed high activity against *C. albicans* and *C. tropicalis.* The synthesized silver nanoparticles using extract of turnip leaf (*Brassica rapa* L.) showed broad-spectrum antifungal activity against wood-degrading fungi such as *Gloeophyllum abietinum, G. trabeum, Phanerochaete sordida*, and *Chaetomium globosum* by inhibiting growth [47]. The high stability of silver nanoparticles and their low chemical reactivity give them a long-lasting activity compared to plant extracts. The plant extracts may display a temporary activity against pathogens, but they are vulnerable to damage after a while due to the presence of different chemical components [48], while after use in the biological manufacture of nanoparticles, the active compounds such as vitamins, amino acids, enzymes, proteins, and alcohol compounds in the extract and responsible for the formation of the nanoparticles act as reducing and covering agents for several months [49]. The effect of the nanoparticles on the cell membrane of the bacterial cell depends on the difference in charges (the result of the difference in charges: negative charge in the cell wall is linked with the positive charge of silver ions) [11]. Size and shape of the particles, as the spherical particles, have a greater inhibition effect compared to the rod-shaped particles, and the small particles can penetrate the cell wall and form free radicals and disrupt cell proteins by the liberated silver ions [45]; after the absorption of silver ions liberated by the pathogenic cell, this leads to disruption of respiratory enzymes and the generation of reactive oxygen species that prevents the formation of ATP, which increases the disruption of the cell membrane. It also works on modifications in the DNA, which caused cell death [50].

**Figure 8:**
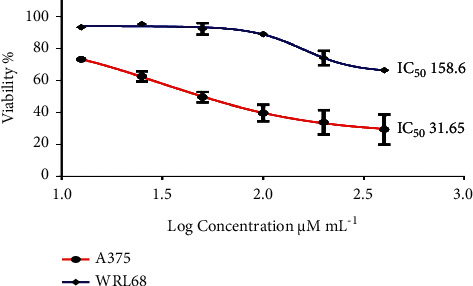
The cytotoxicity of green synthesized silver nanoparticles against A375 human melanoma cell lines and WRL68 normal cell line.

The result of the cytotoxicity test showed that the silver nanoparticles were positively active against the growth of the A375 cell line compared to WRL68 normal cells. The different concentrations of silver nanoparticles solution (12.5–25, 50–100, and 200–400 *μ*g/mL) determined the viability rate of cells, as it showed the ability of silver nanoparticles to prevent the growth of cancer cells in a dose-dependent manner (Figure 8). The effectiveness of the nanoparticles against the A375 cell line was the lowest at 12.5 and 25 *μ*g/mL of concentration which was 73.23% and 62.58%, respectively, and the result of half-maximal inhibitory concentration (IC 50) was 31.65. On the other hand, IC 50 against WRL68 cells was 158.6 *μ*g/mL. The alcoholic extract of *Moringa* leaves with silver was used against the growth of Kasumi-1 leukemia cells and normal myeloid cells (CD34+) [52], and it was selectively cytotoxic to leukemia cells and nontoxic to normal myeloid cells (CD34+) as well as was effective in blocking the cell cycle at G1 and S stages, respectively. In addition, [52] indicated that the plant leaves extract had a positive effect on the melanoma-A2058 cell line, cervical cancer-HeLa cells, and lung cancer-A549 cells, which impeded the cell cycle and caused changes in apoptosis shifts [53]. In contrast, [54] used the aqueous extract of *Moringa* leaves at different concentrations for 48 hours in the cytotoxicity assay using human epithelial carcinoma (KB) cell line and found that it has the potential to stimulate apoptosis and antiproliferative activity that led to an increase in ROS inside the cells and also indicated that the studied plant extract contains polyphenol compounds and flavonoids (quercetin), which act as chemical protective factors in inhibiting the progression of cancer. Humane melanoma cell line represents 10% of skin cancer, and it is rapidly spreading and resistant to many drugs and rarely occurs in the mouth and intestines [55]. It is more common in light-skinned people than dark-skinned people; the causes of its occurrence are not yet known, but it is attributed to DNA damage as a result of exposure to ultraviolet rays or it may be a genetic predisposition [56].

## 4. Conclusion

It has been demonstrated that the aqueous extract of *M. oleifera* leaves is capable of producing AgNPs. The green synthesis (ecofriendly) of silver nanoparticles showed excellent antimicrobial activity against some pathogenic bacteria and fungi (yeast) and possesses significant cytotoxic activity against human melanoma cells A37. This simple procedure for obtaining silver nanoparticles has such an advantage such as compatibility for pharmaceutical and medical applications.

## Figures and Tables

**Table 1:** The inhibition zone for three species (*Candida* and bacteria) of biosynthesized silver nanoparticles by leaves extract of *Moringa oleifera*.

Concentration	Zone of inhibition (mean ± S.E)						*P* value
	*C. albicans*	*C. tropicalis*	*C. glabrata*	*S. aureus*	*E. coli*	*K. pneumoniae*	
Positive control	0	0	0	0	0	0	—
Negative control	0	0	0	0	0	0	—
1000 *μ*g/mL	14.6 ± 0.3	13.0 ± 0.5	18.0 ± 1.0	20.0 ± 0.5	19.0 ± 0.5	14.6 ± 0.6	<0.001
800 *μ*g/mL	13.0 ± 0.8	11.3 ± 0.3	15.3 ± 0.5	18.0 ± 0.5	21.0 ± 0.5	15.0 ± 0.5	<0.001
600 *μ*g/mL	12.3 ± 0.3	12.3 ± 0.3	11.6 ± 0.5	20.6 ± 0.8	21.0 ± 0.5	18.0 ± 0.5	<0.001

Positive control: 1 m·M of AgNO_3_. Negative control: *M. oleifera* leaves extract.

## Data Availability

The data used to support this study are included within the article.
